# Transmission of highly pathogenic avian influenza in the nomadic free-grazing duck production system in Viet Nam

**DOI:** 10.1038/s41598-020-65413-2

**Published:** 2020-05-21

**Authors:** Katriina Willgert, Anne Meyer, Dinh Xuan Tung, Nhu Van Thu, Pham Thanh Long, Scott Newman, Nguyen Thi Thanh Thuy, Pawin Padungtod, Guillaume Fournié, Dirk Udo Pfeiffer, Timothée Vergne

**Affiliations:** 1 Veterinary Epidemiology Economics and Public Health Group, Department of Pathobiology and Population Sciences. The Royal Veterinary College. Hawkshead Lane, North Mymms, Hatfield, Herts AL9 7TA United Kingdom; 2 Disease Dynamics Unit, Department of Veterinary Medicine, University of Cambridge, Madingley Road, Cambridge CB3 0ES United Kingdom; 3 Ausvet Europe, Lyon, 69001 France; 4 National Institute of Animal Sciences, Hanoi, Viet Nam; 5 Institute of Environmental Health and Sustainable Development, Hanoi, Viet Nam; 6 Emergency Center for Transboundary Animal Diseases, Food and Agriculture Organization of the United Nations, Hanoi, Viet Nam; 7 Department of Animal Health, Ministry of Agriculture and Rural Development of Viet Nam, Hanoi, Viet Nam; 8 Centre for Applied One Health Research and Policy Advice, Jockey Club College of Veterinary Medicine and Life Sciences, City University of Hong Kong, Kowloon, Hong Kong, SAR P.R. China; 9 MIVEGEC Group, Institut de Recherche pour le Développement, Montpellier, France; 10 UMR ENVT-INRA 1225, Ecole Nationale Vétérinaire de Toulouse, Toulouse, France

**Keywords:** Influenza virus, Preventive medicine

## Abstract

The presence of free-grazing ducks (FGD) has consistently been shown to be associated with highly pathogenic avian influenza virus (HPAIV) H5N1 outbreaks in South-East Asia. However, the lack of knowledge about the transmission pathways limits the effectiveness of control efforts. To address this gap, we developed a probabilistic transmission model of HPAIV H5N1 in the nomadic FGD production system in Viet Nam, assuming different scenarios to address parameter uncertainty. Results suggested that HPAIV H5N1 could spread within the nomadic FGD production system, with an estimated flock-level effective reproduction number (*r*_*e*_) ranging from 2.16 (95% confidence interval (CI): 1.39-3.49) to 6.10 (95%CI: 3.93-9.85) depending on the scenario. Indirect transmission via boats and trucks was shown to be the main transmission route in all scenarios. Results suggest that *r*_*e*_ could be reduced below one with 95% confidence if 86% of FGD flocks were vaccinated in the best-case scenario or 95% in the worst-case scenario. If vaccination was combined with cleaning and disinfection of transport vehicles twice a week, vaccination coverage could be lowered to 60% in the best-case scenario. These findings are of particular relevance for prioritising interventions for effective control of HPAIV in nomadic free-grazing duck production systems.

## Introduction

Highly pathogenic avian influenza virus (HPAIV) H5N1 is a zoonosis which has resulted in fatal human infections as well as mortality and culling of several hundred million domestic poultry worldwide, with extensive impact on the poultry industry and livelihoods of people globally^1^. Since its emergence in Viet Nam in late 2003, HPAIV H5N1 has been regularly detected in several provinces of the country, demonstrating sustained transmission^2–4^. HPAIV H5N1 outbreaks reported in Viet Nam have often been associated with ducks^5^ and rice production^6,7^. The association between rice production and HPAIV H5N1 occurrence is likely to be a consequence of the management of free-grazing duck (FGD) flocks, which graze on rice fields, and may promote viral spread^8^. Moreover, as infected ducks can be sub-clinically affected, they can facilitate virus persistence within a region and act as a viral reservoir^8,9^.

In Viet Nam, the number of domestic ducks was reported to be between 60^10^ and 69 million^11^ in the early 2010s, with around 18 million located in the Mekong River Delta where 60% of the domestic poultry population of the country is located^10^. There is a wide range of duck farming systems, which commonly overlap^11^. These systems can be categorised into three main groups: (1) confined backyard and commercial ducks, (2) stationary FGD flocks, also called *short-distance* FGD flocks, which feed on rice fields within the village boundaries and are kept on farms overnight and (3) nomadic FGD flocks, also called *moving flocks* or *long-distance* FGD flocks, which are transported over relatively long distances to feed on harvested rice fields and are confined in temporary enclosures at the edge of the rice fields at night^11^. Both stationary and nomadic adult FGDs (>3 months of age) scavenge in flooded rice fields after harvest, feeding on left over grains, insects and molluscs^10^. Nomadic FGD flocks are transported from one grazing place to another mostly by boat but also by truck or on foot^11,12^. Different nomadic FGD flocks can sometimes share the same transport vehicle, and a given vehicle can transport several flocks successively on a single day without being cleaned nor disinfected between journeys. Consequently, nomadic FGD flocks have several opportunities for direct and indirect contacts with other flocks, potentially contributing to the circulation of HPAI viruses. Also, nomadic FGD flocks are regularly transported to different districts and provinces according to the rice production cycle and feed availability^10,12^. Therefore, nomadic FGD flocks are part of a highly connected network of long-distance movements, where a single journey can be more than 100 kilometres. As an example, in southern Viet Nam, 68% and 33% of duck grazing sites are located outside of the commune and province of residence of the farmer, respectively^12^. For these reasons, long-distance FGD flocks are suspected to play a significant role in the maintenance and spread of avian influenza viruses (AIV)^13^.

The objectives of this study were (1) to assess the extent to which the nomadic FGD production system can contribute to HPAIV H5N1 spread, (2) to estimate the relative contribution of different transmission routes within this production system and (3) to evaluate the effectiveness of potential preventive measures to decrease the risk of viral transmission. To meet these objectives, a probabilistic disease transmission model was designed and parameterised based on data generated through interviews with stakeholders, field observations and published literature.

## Methods

### Overview of the probabilistic transmission model

A probabilistic disease transmission model was developed to estimate the effective reproduction number (*r*_*e*_) defined as the average number of nomadic FGD flocks that would be infected by one HPAIV H5N1 infected nomadic FGD flock over the course of its infectious period, in a population initially composed of either susceptible or vaccinated flocks. Note that *r*_*e*_ differs from the basic reproduction number (*R*_0_), as *r*_*e*_ accounts for a proportion of the population being vaccinated, where vaccinated flocks are assumed to be protected against infection. If the vaccination coverage is null, all flocks are assumed to be susceptible to HPAIV H5N1 so that *r*_*e*_ = *R*_0_.

Several pathways of exposure were considered to account for the diversity of transmission routes between nomadic FGD flocks. In April 2016, a risk assessment workshop was held in Hanoi, Viet Nam, to identify the most relevant transmission routes, and to discuss potential control strategies that could be implemented to reduce the risk of HPAIV H5N1 transmission in the nomadic FGD production system. It was attended by 30 stakeholders, including members of the regional animal health offices of the Department of Animal Health of the Ministry of Agriculture and provincial authorities, representatives of the Food and Agriculture Organization of the United Nations (FAO), and researchers. Six transmission routes were selected as the most relevant: (1) direct and (2) indirect contact while grazing on a rice field, (3) direct and (4) indirect contact during boat transportation from one grazing site to another, (5) direct and (6) indirect contact during truck transportation from one grazing site to another.

The effective reproduction number (*r*_*e*_) of HPAIV H5N1 in the nomadic FGD production system was the sum of the effective reproduction numbers across the six different transmission routes:

$${r}_{e}=\mathop{\sum }\limits_{w=1}^{6}\,{r}_{e\_w}$$

with ${r}_{e\_w}$ being the average number of transmission events over the course of the infectious period of an infected nomadic FGD flock via a particular transmission route *w*, defined by

$${r}_{e\_w}={p}_{w}\,\ast \,{c}_{w}\,\ast \,{g}_{cycle}$$

with *p*_*w*_ being the probability of a susceptible nomadic FGD flock becoming infected given it has been in contact with an infected nomadic FGD flock through transmission route *w*, $\,{c}_{w}$ being the average number of nomadic FGD flocks with which a given nomadic FGD flock got into contact through the transmission route *w* during one grazing cycle (*i.e*. from first release in a grazing site to the first release in the next grazing site), and ${g}_{cycle}$ being the average number of grazing cycles undergone by an infected nomadic FGD flock during its infectious period. Most nomadic FGD flocks consist of layer ducks for which the production cycle can last up to two years (Meyer *et al*., 2017). Consequently, it was assumed that the life expectancy of ducks in a flock was much longer than the average infectious period of the flock and ${g}_{cycle}$ was not affected by the replacement of ducks within flocks. It was also assumed that two given nomadic FGD flocks only come into contact through a single transmission route over the study period (i.e. the duration of the flock-level infectious period which is described in supplementary material).

### Probabilistic formulation of each transmission route

#### Transmission by direct contact in a rice field

It was assumed that transmission of HPAIV H5N1 by direct contact between two nomadic FGD flocks grazing on neighbouring rice fields at the same time could occur through two events: (i) infected ducks from an infected flock that temporarily joined a susceptible flock transmitted the infection to at least one duck of the susceptible flock (with a probability *p*_*1a*_) or (ii) at least one duck from a susceptible flock that temporarily joined an infected flock became infected (with a probability *p*_*1b*_). For a given direct contact opportunity, these two events were considered mutually exclusive. Therefore, the probability of transmission between two flocks given a direct contact in the field (*p*_*1*_) was expressed as

$${p}_{1}=1-{(1-s\ast ({p}_{1a}+{p}_{1b}))}^{\alpha }$$

with α being the average number of times ducks from a flock temporarily joined another flock that grazed on a neighbouring rice field during one grazing period, *s* being the probability that the non-infectious nomadic FGD flock was susceptible to HPAIV H5N1 (i.e. unvaccinated) and *p*_*1a*_ and *p*_1*b*_ being the probabilities that at least one duck from the susceptible flock becomes infected when visiting ducks were from the infectious flock and susceptible flock, respectively. These two latter probabilities were expressed as follows:

$${p}_{1a}=\gamma \,\ast \,(1-{(1-\pi \ast \delta )}^{n})$$

$${p}_{1b}=(1-\gamma )\,\ast (1-{(1-\varepsilon )}^{n})$$

with *γ* being the probability that the visiting ducks were from the infectious flock, *π* being the average prevalence of HPAIV H5N1 infected ducks in the infectious flock, *δ* being the probability that an infectious duck which temporarily joined a susceptible flock infected at least one susceptible duck, *n* being the number of ducks that temporarily joined the other flock. Therefore, $1-{(1-\pi \ast \delta )}^{n}$ was the probability that at least one duck from an infectious flock which temporarily joined a susceptible flock infected at least one duck in the susceptible flock. The probability that the visiting ducks were from the susceptible flock was described by $(1-\gamma )$, *ε* was the probability that a susceptible duck which temporarily joined the infectious flock became infected, so $1-{(1-\varepsilon )}^{n}$ was the probability that at least one susceptible duck which temporarily joined the infectious flock became infected.

The average number of nomadic FGD flocks that can come into direct contact with a given flock in a rice field during one grazing cycle (c_1_) was assumed to be equal to the average number of nomadic FGD flocks that graze in adjacent fields.

#### Transmission by indirect contact in a rice field

Since HPAIV H5N1 has been shown to survive for several days in the environment, water and faeces^14–16^, transmission of HPAIV H5N1 could also occur between two nomadic FGD flocks that successively graze on the same harvested rice field before the start of a new rice production cycle. Consequently, a susceptible nomadic FGD flock could become infected if it visited a grazing site which was previously visited by a HPAIV H5N1 infected nomadic FGD flock within the time frame of the virus survival period. Given that a maximum of two flocks could graze successively at the same grazing site before the new rice production cycle starts again (field observation), the probability of transmission given an indirect contact in a field (*p*_2_) was calculated as follows:

$${p}_{2}=\theta \,\ast \,s\,\ast \,\lambda $$

with *θ* being the probability that the first visiting FGD flock was the infectious one, *s* being the probability that the second visiting FGD flock was susceptible to HPAIV H5N1 (i.e. unvaccinated) and *λ* being the probability that at least one duck from the susceptible flock became infected following exposure to the virus on a contaminated rice field.

Domanska-Blicharz *et al*.^17^ established experimentally that H5N1 HPAIV could remain infective in pond water at 20 °C for about 14 days and that its survival rate decreased with increasing temperature. From 2005 to 2014, the mean daily temperature in the Mekong Delta region was 26.4-27.9 °C^18^. Thus, assuming that natural conditions are more detrimental to virus survival than experimental conditions, we considered that the average infectious survival period in a flooded rice paddy was seven days. Again, given that a maximum of two flocks could graze successively at the same grazing site before the new rice production cycle starts again, the average number of nomadic FGD flocks that visit a grazing site within seven days after a first FGD flock has left the grazing site (c_2_) was calculated as follows:

$${c}_{2}=\eta \,\ast \,\kappa $$

with $\eta$ being the probability that two nomadic FGD flocks grazed successively on the same rice paddy field during the same production cycle and *κ* being the probability that the second flock arrived within seven days after the first flock had gone.

#### Transmission by direct contact during transportation in boats

When being moved by boat from a grazing location to the next, a nomadic FGD flock can be transported with other nomadic FGD flocks. Even though the flocks are kept on different floors in the boats, this setting may promote viral transmission between flocks since contaminated dust, feathers and equipment can be easily moved between floors^12^. Consequently, the probability of transmission given a direct contact in a boat (*p*_3_) was calculated as follows:

$${p}_{3}=s\,\ast \,{o}_{boat}$$

with *s* being the probability that the non-infectious nomadic FGD flock was susceptible to HPAIV H5N1 (i.e. unvaccinated) and *ο*_*boat*_ being the probability that at least one duck from a susceptible nomadic FGD flock became infected if it was transported with an infectious flock in the same boat.

Given that only one transportation event occurs per grazing cycle and that most transportation events involved only one or two flocks (field observation), the average number of other flocks a given flock was concurrently transported with on a boat during a grazing cycle was estimated as

$${c}_{3}={\mu }_{boat}\,\ast \,{\xi }_{boat}$$

with *μ*_*boat*_ being the probability that a nomadic FGD flock was transported to another grazing site by boat (as opposed to by truck or by foot) and *ξ*_*boat*_ being the probability that two nomadic FGD flocks were transported together on the same boat.

#### Transmission by indirect contact during transportation on boats

Since, HPAIV H5N1 can survive for several days in the environment^19,20^, a susceptible nomadic FGD flock could become infected if it is transported on a contaminated boat which previously transported an infectious FGD flock within the time window corresponding to the virus survival period if the boat had not been disinfected yet. Consequently, the probability of transmission given an indirect contact on a boat (*p*_4_) was calculated as follows:

$${p}_{4}=s\ast {\tau }_{boat}$$

with *s* being the probability that the nomadic FGD flock that was transported on a boat subsequently to an infectious nomadic FGD flock was susceptible to HPAIV H5N1 (i.e. unvaccinated) and τ_boat_ being the probability that at least one duck from the susceptible nomadic FGD flock became infected during transport if the boat was contaminated.

The average number of indirect contacts an infectious nomadic FGD flock had with susceptible flocks on a boat (c_4_) was based on the average number of nomadic FGD flocks that were transported before the boat was cleaned and disinfected or the excreted virus was deactivated. Consequently, it was defined by

$${c}_{4}={\mu }_{boat}\,\ast \,{\varphi }_{boat}\,\ast \,{\sigma }_{boat}\,\ast \,in{f}_{boat}$$

with μ_boat_ being the probability that the infectious flock was transported by boat, ${\varphi }_{boat}$ being the average number of flocks transported per journey (${\varphi }_{boat}={\xi }_{boat}+1)$, ${\sigma }_{boat}$ being the average daily number of boat journeys, and *inf*_*boat*_ being the number of days the environment of the boat remained infectious. To account for the frequency of cleaning and disinfection, *inf*_*boat*_ was calculated as follows:

$$in{f}_{boat}=\,{\rm{\min }}(surv,\,tc{d}_{boat})$$

with *surv* being the time period the virus survived in the environment and *tcd*_*boat*_ being the average length of time until the next cleaning and disinfection of the boat. Given that duck transportation could happen anytime between two cleaning and disinfection events, *tcd*_*boat*_ was defined by *tcd*_*boat*_ = 0.5*ρ_*boat*_*, with ρ*_*boat*_ being the average number of days between two cleaning and disinfection events in a boat.

#### Transmission by direct and indirect contact during transportation on trucks

To define the probabilities of transmission given direct and indirect contacts on trucks (*p*_5_ and *p*_6_ respectively) as well as the number of direct and indirect contacts an infectious nomadic FGD flock had on a truck with susceptible flocks during a single grazing cycle, (*c*_5_ and *c*_6_ respectively), the formulations used for direct and indirect contacts on boats were adapted with truck-specific probabilities.

### Model parameterisation

Most of the model parameter values were informed by a field observational survey conducted with FGD farmers, rice field owners and FGD transporters. This data was collected during face-to-face interviews held between October and December 2015 in the Mekong Delta region where FGD farming is most prevalent, described in detail in Meyer *et al*.^12^. Corresponding parameters were associated with appropriate probability distributions to capture interviewees’ response variability. Most of the other parameter values were adapted from information in published literature. The seven probabilities of infection given exposure (i.e. *δ, ε, λ, ο*_*boat*_*, τ*_*boat*_*, ο*_*truck*,_ *τ*_*truck*_) could not be estimated in a straightforward manner. They were drawn from ranges of plausible values defined by a semi-quantitative assessment based on expert judgement (see section on sensitivity analysis). All model parameters are presented in Table 1, along with their values or distributions and associated references. Note that parameters related to control strategies (marked with an asterisk in Table 1) were given fixed values to facilitate comparison of the effectiveness of different strategies.

**Table 1:** Parameters and input values used in the baseline scenario.

Description	Parameter	Value	Reference
Within-flock transmission rate parameter	*β*	Pert(0.5,0.8,1.2)	^28^
Average duration of the infectious period for an infected duck	*r*	Pert(2,3,4)	^28^
Within-flock prevalence of HPAIV H5N1	*π*	Transmission model	Supplementary Material
Average number of grazing cycles undergone by an infected nomadic FGD flock during its infectious period	*g*_*cycle*_	Transmission model	Supplementary Material
Average number of times ducks from a nomadic FGD flock temporarily escape into a neighbouring flock during a grazing cycle	*α*	Gamma(20,15)	Field data (F)
Probability that the ducks that are temporarily integrated into the other flock in the field are from the infectious flock	*γ*	0.5	NA
Average number of ducks that temporarily escape into another flock	*n*	Pert (1, 5, 20)	NA
Probability that a FGD flock is susceptible to HPAIV H5N1 (i.e. unvaccinated)	s*	0.5	DAH, pers. com.
Probability that an infected duck that has temporarily joined a susceptible flock infects at least one susceptible duck	*δ*	[0.1 – 0.6]	NA
Probability that a susceptible duck that has temporarily joined an infectious flock becomes infected	*ε*	[0.1 – 0.6]	NA
Probability that two FGD flocks graze successively on the same rice paddy field during the same production cycle	*η*	Beta (13, 9)	field data (RPO)
Probability that the infectious FGD flock is the first to visit the rice paddy field	*θ*	0.5	NA
Probability that a nomadic FGD flock arrives within 7 days after the departure of the previous flock	*κ*	Beta (9,3)	field data (RPO)
Probability that the susceptible flock becomes infected following exposure to environmental contamination with HPAIV H5N1 in the rice paddy field	*λ*	[0.1 – 0.6]	NA
Probability that a FGD flock is transported to another grazing site by boat	*μ*_*boat*_	Beta (19,4)	field data (F)
Probability that two flocks are transported together in the same boat	*ξ*_*boat*_	0.31	field data (T)
Probability that at least one duck from the susceptible FGD flock becomes infected during transport if it is transported together with an infectious flock in the same boat	*ο*_*boat*_	[0.6 – 1.0]	NA
Probability at least one duck from the susceptible FGD flock becomes infected during transport if the boat is contaminated	*τ*_*boat*_	[0.2 – 0.6]	NA
Time period the transportation vehicle remains infectious	*inf*_*boat*_	min(surv, tcd_boat_)	NA
Average number of daily journeys for boats	*σ*_*boat*_	1.325	field data (T)
Average number of flocks transported per boat journey	*φ*_*boat*_	1.31	field data (T)
HPAIV H5N1 environmental survival in a transport vehicle (days)	*surv*	Pert(2,5,7)	Adapted from^19^
Average length of time until the next cleaning and disinfection of a boat (days)	*tcd*_*boat*_	0.5*ρ_*boat*_	NA
Average duration between two cleaning and disinfection events of vehicle (days)	*ρ*_boat_*	30	field data (T)
Probability that a FGD flock is transported to another grazing site by truck	*μ*_*truck*_	Beta (10,36)	field data (F)
Probability that two flocks are transported together in the same truck	*ξ*_*truck*_	0.31	field data (T)
Probability at least one duck from the susceptible FGD flock becomes infected during transport if it is transported together with an infectious flock in the same truck	*ο*_*truck*_	[0.6 – 1.0]	NA
Probability at least one duck from the susceptible FGD flock becomes infected during transport if the truck is contaminated	*τ*_*truck*_	[0.2 – 0.6]	NA
Time period the transportation vehicle remains infectious	*inf*_*truck*_	min(surv, tcd_truck_)	NA
Average number of daily journeys for trucks	*σ*_*truck*_	3	field data (T)
Average number of flocks transported per truck journey	*φ*_*truck*_	1.31	field data (T)
Average length of time until the next cleaning and disinfection of a truck (days)	*Tcd*_*truck*_	0.5*ρ_*truck*_	NA
Average duration between two cleaning and disinfection events of vehicle (days)	*ρ*_truck_*	10.6	field data (T)

Field data are unpublished data derived from field interviews; RPO = rice paddy owner; F = nomadic free grazing duck flock farmer; T = nomadic free-grazing duck flock transporter; NA = not applicable; parameters with an asterisk were given fixed values to simplify the comparison between the alternative control strategies.

The probability distribution of the average number of grazing cycles included in an infectious period of a FGD flock (${g}_{cycle}$) and the average within-flock prevalence (π) were determined by running Monte Carlo simulations of a frequency-dependent deterministic transmission model (see details in the supplementary material). The infectious period of a FGD flock was defined as the period between viral incursion in the flock and the time at which the average proportion of infected ducks fell below 0.01.

To our knowledge, no published data were available for the average number of ducks escaping temporarily from their flock to join another one while grazing in a field (*n*). Because FGD farmers never reported more than 20 ducks escaping their flocks, this parameter *n* was associated with a Pert distribution with the parameters set to 1 (minimum), 5 (most likely) and 20 (maximum) ducks.

HPAIV H5N1 can remain infectious for several days in faeces or water. Kurmi *et al*.^19^ estimated a survival time in dry and wet faeces of 5 days at 24 °C, while according to Phong^21^, HPAIV H5N1 can survive in chicken manure for 7 days at 20 °C. Therefore, we assumed that the average infectious survival period in transport vehicles (*surv*) was around 5 days and this parameter was assigned a Pert distribution with values 2 (minimum), 5 (most likely) and 7 (maximum) days.

### Sensitivity analysis

Because of the uncertainty associated with the probability that a susceptible duck temporarily joining an infectious flock becomes infected (*ε*) and the probability that an infectious duck temporarily joining a susceptible flock infects at least one susceptible duck (*δ*), both parameters were assumed to range from very low to high (between 0.1 and 0.6). In the estimation of the overall effective reproduction number (*r*_*e*_), both parameters were therefore given the same probability of transmission given exposure. The probability that at least one duck from a susceptible flock becomes infected following exposure to infectious virus in a contaminated rice field (*λ*) was also considered very low to high (between 0.1 and 0.6). The probabilities that at least one duck from a susceptible flock becomes infected given it was transported together with an infectious flock on a boat or a truck (*ο*_*boat*_ and *ο*_*truck*_) were considered higher than *ε* and *δ* due to the close proximity between ducks during a relatively long period of time (usually several hours) and therefore assumed to range from high to very high (between 0.6 and 1). Finally, the probabilities that at least one duck from the susceptible FGD flock becomes infected during transport if the boat or truck (*τ*_*boat*_ and *τ*_*truck*_, respectively) were contaminated were assumed to range from low to high (between 0.2 and 0.6). Note that these probabilities of indirect transmission in transport vehicles had a higher lower bound than those of indirect transmission in the field (*ε* and *δ*) because of a higher density of birds in the vehicles as well as a higher expected concentration of viruses in the transport vehicles than in the flooded rice fields.

To assess how the uncertainty in some parameter values influenced model outputs, the value of each of these seven parameters (*δ, ε, λ, ο*_*boat*_*, τ*_*boat*_*, ο*_*truck*,_ τ_truck_) was changed individually with step increments of 0.1 within their likely range. For each respective value of the seven parameters, 10,000 simulations were run. The impact of the uncertainty associated with these seven parameters was assessed using two model outputs: the effective reproduction number *r*_*e*_ and the relative contribution of the six transmission routes to *r*_*e*_. All simulations and analyses were performed using the R software version 3.3^22^.

### Effectiveness of alternative control strategies

The effectiveness of three potential control strategies identified by local and national stakeholders during the previously mentioned workshop were assessed. These included improved vaccination coverage (defined by parameter *s*), and increased frequency of cleaning and disinfection of boats and trucks (defined by parameters *ρ*_*boat*_ and *ρ*_*truck*_, respectively). The impact of these three strategies was evaluated by changing the value of these parameters and running 10,000 Monte-Carlo simulations for each parameter set.

### Ethical statement

This study did not involve any animal experiment.

## Results

When accounting for all transmission routes and assuming a flock-level vaccination coverage of 50%, the average number of susceptible nomadic FGD flocks that would be infected by one HPAIV H5N1 infectious nomadic FGD flock over the course of its infection (*r*_*e*_) was estimated to be 2.16 [95% confidence interval (CI): 1.39-3.49] for the overall best-case scenario (considering minimal values for the seven transmission probabilities given exposure) and 6.10 [95%CI: 3.93-9.85] for the overall worst-case scenario (considering maximal values for the seven transmission probabilities given exposure).

**Figure 1:**
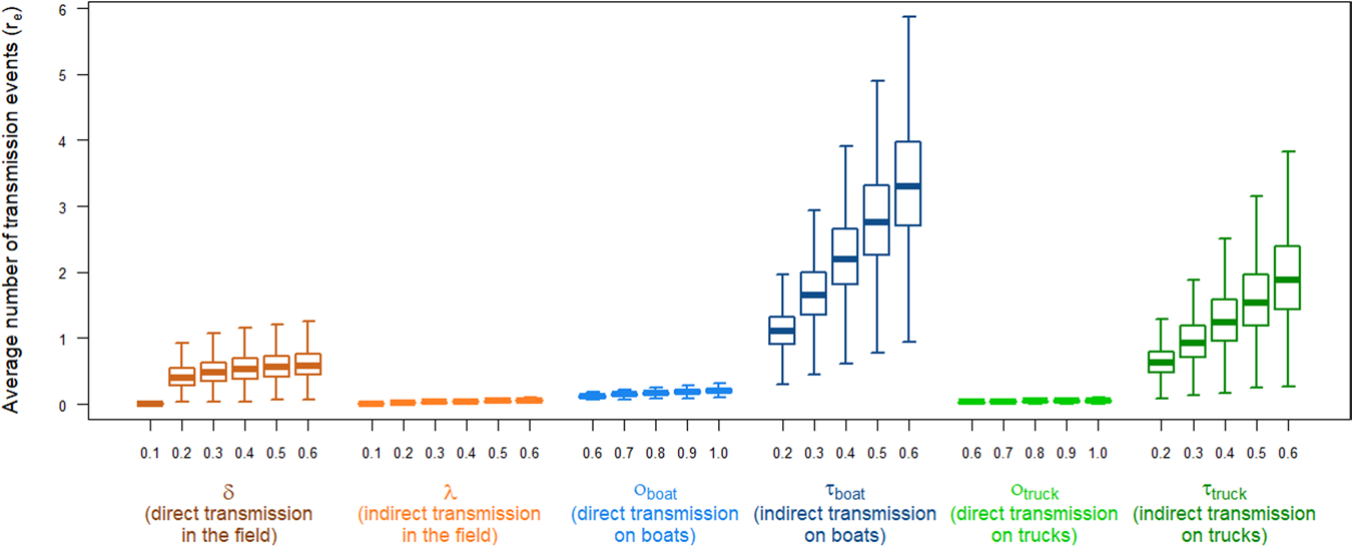
Distributions of the average number of susceptible nomadic FGD flocks that would be infected by one HPAIV H5N1 infectious nomadic FGD flock over the course of its infection through each of the six most likely transmission routes and different probabilities of transmission given exposure.

As shown in Fig. 1, the model suggests that indirect transmission in the field as well as direct transmission on boats or trucks contribute only marginally to the transmission of avian influenza in the nomadic FGD production system. Indeed, their corresponding effective reproduction number *r*_*e*_ under their worst-case scenarios was smaller than the effective reproduction number for the three other transmission routes under their best-case scenarios (Fig. 1).

Assuming that the probability of at least one duck from the susceptible FGD flock becoming infected during transportation on contaminated boats (*τ*_*boat*_) is of the same magnitude as on contaminated trucks (*τ*_*truck*_), indirect transmission on boats appears to contribute more substantially to the overall transmission of HPAIV H5N1 in the nomadic FGD production system than indirect transmission on trucks. As illustrated in Fig. 1, for a given equal value of *τ*_*boat*_ and *τ*_*truck*_, the distribution of *r*_*e*_ by indirect transmission on boats (dark blue) is higher than *r*_*e*_ by indirect transmission on trucks (dark green).

**Figure 2:**
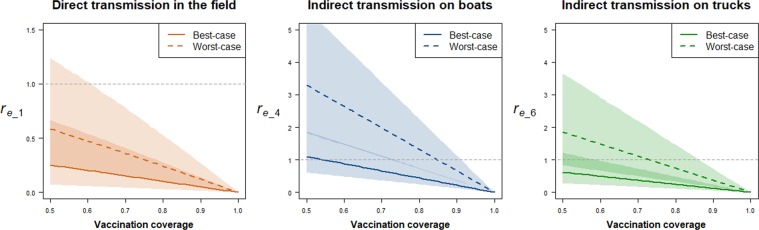
Impact of an increase in vaccination coverage on the average number of susceptible nomadic FGD flocks that would be infected by a HPAIV H5N1 infectious nomadic FGD flock over the course of its infection by direct transmission in the field (left), indirect transmission on boats (middle) and trucks (right). Lines represent medians and coloured polygons represent the 95% confidence regions.

The effect of an increase in the vaccination coverage above the assumed 50% was evaluated for the three main transmission routes (i.e. direct transmission in the field and indirect transmission on boats and trucks). In the best-case scenario, ensuring that the expected numbers of indirect transmission events occurring on boats (*r*_*e_4*_) and trucks (*r*_*e_6*_) remain below 1 with 95% confidence requires at least 74% and 59%, respectively, of FGD flocks to be vaccinated and fully protected (Fig. 2). A higher vaccination coverage would be required on boats compared to trucks due to the effective reproduction number (*r*_*e*_) estimated for the individual transmission routes being greater on boats. With 70% of flocks being vaccinated, the overall effective reproduction number (*r*_*e*_) would still be higher than 1, ranging from 1.30 [95%CI: 0.84-2.11] for the overall best-case scenario to 3.67 [95%CI: 2.37-5.92] for the overall worst-case scenario. According to the overall best-case (respectively worst-case) scenario, ensuring that *r*_*e*_ < 1 with 95% confidence would require 86% (resp. 95%) of flocks to be vaccinated.

**Figure 3:**
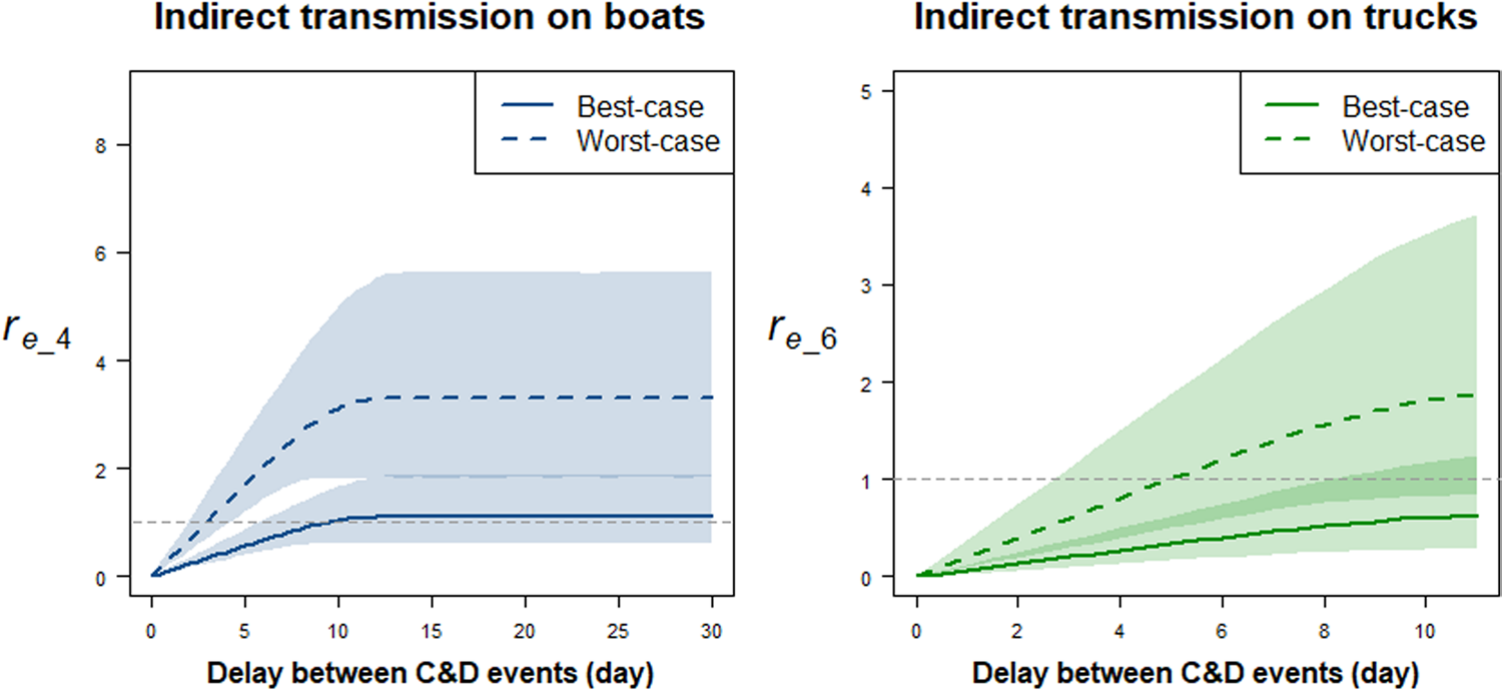
Impact of variations in the length of time between successive cleaning and disinfection (C&D) of transport vehicles on the average number of susceptible nomadic FGD flocks that would be infected by a HPAIV H5N1 infectious nomadic FGD flock over the course of its infection by indirect transmission in boats (left) and trucks (right). Lines represent medians and coloured polygons represent the 95% confidence regions.

Figure 3 illustrates the effect of decreasing the length of time between successive cleaning and disinfection events on boats (from 30 to 0 days) and trucks (from 11 to 0 days) on *r*_*e_4*_ and *r*_*e_6*_, respectively, with a vaccination coverage of 50%. For both types of transport vehicles and considered parameter scenarios, cleaning and disinfection would only have a substantial impact on transmission if implemented at least once every 10 days. In their respective best-case scenario, reducing *r*_*e_4*_ and *r*_*e_6*_ below 1 with 95% confidence requires boats to be cleaned and disinfected at least every six days and trucks every eight days (Fig. 3). With boats and trucks being cleaned and disinfected every six and eight days, respectively, the overall effective reproduction number (*r*_*e*_) would still be higher than 1, and estimated to range between 1.33 [95%CI: 0.91-2.10] for the overall best-case scenario and 3.88 [95%CI: 2.66-6.14] for the overall worst-case scenario. In the overall best-case scenario, the simulations suggested that cleaning and disinfecting boats and trucks every day would just be sufficient to ensure that *r*_*e*_ is below 1 with 95% confidence.

**Figure 4:**
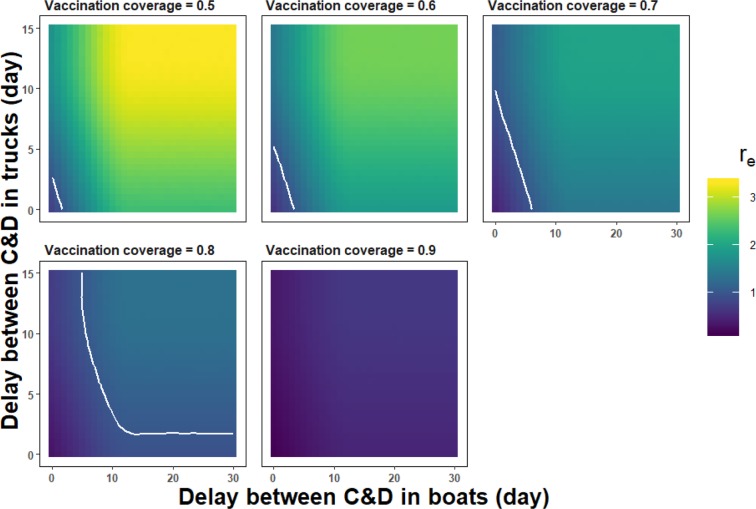
Impact of different combinations of vaccination coverage and length of time between successive cleaning and disinfection (C&D) of transport vehicles on the average number of susceptible nomadic FGD flocks that would be infected by a HPAIV H5N1 infectious nomadic FGD flock over the course of its infection (*r*_*e*_) in the best-case scenario. The colour scale illustrates the 95^th^ percentile of the distribution of *r*_*e*_. The white line represents the limit of the 95% confidence that *r*_*e*_ < 1.

Combining increased vaccination coverage with increased frequency of cleaning and disinfection of boats and trucks could be a feasible alternative to using one of these three interventions alone. Figure 4 illustrates the impact a combination of these three strategies would have on the 95^th^ percentile of the overall *r*_*e*_ for the best-case scenario. Ensuring that *r*_*e*_ < 1 with 95% confidence could be achieved with a vaccination coverage around 80% if boats and trucks were cleaned and disinfected at least once a week. The same results could be obtained with a vaccination coverage around 60% if boats and trucks were cleaned and disinfected at least twice a week. In the worst-case scenario (results not represented), the vaccination coverage would need to exceed 80% and boats and trucks be cleaned and disinfected every day.

## Discussion

In this study, a probabilistic disease transmission model was developed to estimate the effective reproduction number (*r*_*e*_) associated with the transmission of HPAIV H5N1 in nomadic FGD flocks in Viet Nam and quantify the effect of alternative intervention strategies on *r*_*e*_. In a nomadic FGD population with 50% of flocks being vaccinated, one HPAIV H5N1 infectious nomadic FGD flock would, on average, infect 2.16 [95%CI: 1.39-3.49] susceptible nomadic FGD flocks over the course of infection in a best-case scenario and 6.12 [95%CI: 3.93-9.85] FGD flocks in a worst-case scenario. Given that HPAIV H5N1 infection rarely causes mortality in ducks in the Mekong Delta region (Nguyen *et al*., 2014), the relatively high value of *r*_*e*_ suggests that HPAIV H5N1 could spread within the nomadic FGD production system, eventually leading to a high seroprevalence in nomadic FGD flocks. To our knowledge, no serological survey of HPAIV H5N1 infection in nomadic FGD flocks contemporary to our study is available to test this hypothesis. However, it supports the outcomes of a survey conducted in south Viet Nam in 2007-2008 where 42.6% (95% CI: 38.0 – 47.2) of unvaccinated FGD flocks were estimated to be seropositive for H5 despite the absence of suspected mortality^23^.

Transmission through indirect contacts between flocks during transportation in boats or trucks were found to be the main transmission routes. Although increasing the vaccination coverage or frequency of vehicle cleaning and disinfection alone were shown to be effective in reducing disease transmission, this would require a high vaccination uptake or, alternatively, daily cleaning and disinfection of vehicles in best-case scenarios. Vaccination affects all transmission routes by decreasing the probability of an in-contact nomadic FGD flock being susceptible. If vaccination was used alone, the minimum vaccination coverage required to reduce *r*_*e*_ to less than one was 86% in a best-case scenario or 95% in a worst-case scenario. A study in the Mekong Delta of Viet Nam found that the odds of a HPAIV H5N1 outbreak occurring was highest in unvaccinated flocks, intermediate in flocks vaccinated once, and lowest in flocks vaccinated at least twice^24^. In this study, within-flock vaccination coverage was not considered and vaccinated flocks were assumed to be fully protected against HPAIV H5N1. Cuong *et al*.^25^ showed that, in vaccinated flocks, the proportion of ducks (mostly confined ducks and stationary FGD) that were vaccinated twice was as low as 2.8% in small flocks and 31.8% in large flocks, questioning the effectiveness of vaccination at flock level. In addition, vaccination campaigns are particularly challenging in the context of nomadic FGD flocks since the vaccination protocol consists of two injections at a 3-week interval, while nomadic FGD flocks rarely stay more than four weeks at the same grazing location. As a consequence, most vaccinated nomadic FGD flocks are only vaccinated once, resulting in incomplete protection. Therefore, the proportion of FGD flocks which are vaccinated twice is expected to be even lower than that reported in Cuong *et al*.^25^, meaning that, in this study, we may have overestimated the proportion of flocks vaccinated and protected against infection and, therefore, underestimated *r*_*e*_. Consequently, vaccination protocols for nomadic FGD flocks should be improved by promoting inter-provincial collaborations of veterinary services in order to increase the vaccination coverage of nomadic FGD flocks.

Assuming that the challenges associated with achieving adequate vaccination of nomadic FGD flocks can be addressed, increasing vaccination uptake together with improved hygiene practices in transport vehicles may be a more feasible control strategy to reduce indirect exposure of nomadic FGD flocks to HPAIV H5N1. Our results suggest that if vaccination was combined with weekly (respectively twice weekly) cleaning and disinfection of transportation vehicles, the vaccination coverage at flock level required to achieve *r* < 1 with 95% confidence in the best-case scenario could be reduced to 80% (respectively 60%). A certification scheme promoting “clean transport vehicles” could be developed to allow FGD farmers to select transportation vehicles with a lower infection risk and reward transporters who commit to cleaning and disinfecting their transport vehicles on a regular basis. Such an incentive system would need to be fully supported by both transporters and FGD farmers. If successful, one could expect a shift amongst transporters towards good hygiene practices, thereby leading to a decreased risk of transmission of AIV through indirect contact during transport.

Transporting several flocks together on the same vehicle is mostly practiced by owners of small nomadic FGD flocks (<1000 ducks) in order to reduce transport costs. This practice is expected to result in direct contacts between different flocks and, therefore, promote the spread of AIVs between flocks. However, the model suggests that this transmission route may only play a marginal role in AIV transmission due to the small number of susceptible flocks that would be exposed via this route compared to the number of flocks indirectly exposed through contaminated transport vehicles. Therefore, discouraging the transport of more than one flock per vehicle is unlikely to substantially reduce the overall probability of AIV transmission.

The transmission pathways considered in the assessment are unlikely to explain all HPAIV H5N1 cases occurring in nomadic FGD flocks. The transmission routes included in the study were those perceived as most important in the Mekong Delta region^12^. Indirect contact between duck flocks grazing simultaneously at two adjacent sites, resulting from flooding of rice crop fields and water flow, was excluded due to the high uncertainty associated with this risk pathway and its parameter values. Other possible routes of transmission include, but are not limited to, introduction of replacement stock into an existing flock, indirect contact mediated by human visitors and wildlife, contact between two flocks when swimming in the waterways, and contamination of waterways by contaminated wastewater and material such as bird carcasses and manure. In the An Giang province of the Mekong Delta region, 100% of farmers keeping nomadic FGD flocks mentioned ducks had contact with duck traders, 58% reported ducks had contacts with veterinarians during vaccination, 46% with visitors, and 69% with other ducks or chicken. The frequency of these contacts ranged from two to four times per production cycle, the exception being the laying period during which egg traders would visit twice a week^21^. Since these potential transmission routes were not accounted for in our study, *r*_*e*_ is likely to be underestimated and alternative transmission routes would require further assessment in future studies.

The probability of infection occurring in a susceptible FGD flock following exposure to HPAIV H5N1 depends on the type of exposure (direct or indirect), transmission route, contact rate, the infectious dose and host factors such as species and immune status. As a result, there is a high uncertainty associated with any estimate of the probability of infection following exposure, justifying the sensitivity analysis presented in Fig. 1. Previous exposure to AIVs of the same HA subtype could also reduce the susceptibility to HPAIV H5N1^26^. Nevertheless, the duration of immunity to new homologous and heterologous AIV subtypes following infection needs to be further investigated^26^.

A limited number of control strategies were considered in this study. Additional interventions could include health assessment and quarantine of ducks before movement, minimizing direct contact between FGD flocks by using nets, double fencing and avoiding co-grazing, appropriate disposal of carcasses and manure, biosecurity and protective personal equipment for workers visiting FGD flock sites, sanitation of traders’ equipment between farms, and education for early recognition of disease and intervention.

Biosecurity implementation can be challenging in certain farming systems^27^. When enforcement of biosecurity is impractical, vaccination becomes one of the main control measures available^27^, which was highlighted in this study. The results can be used to examine strategies to tackle avian influenza in nomadic FGD populations and prioritise control methods based on their impact and feasibility.

## Supplementary information

Supplementary information.
