# Supplementary material for: Transmission of highly pathogenic avian influenza in the nomadic free-grazing duck production system in Viet Nam
Source: Sci Rep. 2020 May 21;10:8432. doi: 10.1038/s41598-020-65413-2 (PMC7242457; doi:10.1038/s41598-020-65413-2)
Supplement: Supplementary file 1 — Supplementary information. [file 41598_2020_65413_MOESM1_ESM.docx]

**SUPPLEMENTARY MATERIAL**

**Transmission of highly pathogenic avian influenza in the nomadic free-grazing duck production system in Viet Nam**

Katriina Willgert, Anne Meyer, Dinh Xuan Tung, Nhu Van Thu, Pham Thanh Long, Scott Newman, Nguyen Thi Thanh Thuy, Pawin Padungtod, Guillaume Fournié, Dirk Udo Pfeiffer and Timothée Vergne

The probability distribution of the average number of grazing cycles included in an infectious period of a FGD flock ($g_{cycle}$) and the average within-flock prevalence (π) were determined by simulating disease transmission using a frequency-dependent deterministic transmission model. Briefly, ducks could pass through three successive and mutually exclusive states: susceptible (S), infectious (I) or recovered (R). The dynamic of the number of ducks in each state was modelled using the following system of differential equations:

$$\left\{ \begin{aligned} \frac{dS}{dt}=-\beta\frac{S*I}{N} \\ \frac{dI}{dt}=\beta\frac{S*I}{N}-rI \\ \frac{dR}{dt}=rI \end{aligned} \right.$$

with *β* being the transmission rate, S and I being the number of susceptible and infectious ducks, respectively, and N being the total number of ducks in the flock. Ducks remained infectious during a period averaging the infectious period duration (1/*r*) after which they became recovered (R). The values of parameters *β* and *r* were sampled from corresponding Pert distributions to account for associated uncertainty (Table 1). This transmission model assumed that ducks did not die from the disease, so that *N* remained constant. Since the model was run deterministically, the infectious period of a flock was defined as the period between viral incursion and the time at which the average proportion of infected ducks fell below 0.01. The average number of grazing cycles undergone by an infected nomadic FGD flock during its infectious period (*g_cycle_*) was calculated by averaging the ratio between the duration of the infectious period of the flock (in days) by the average duration of a grazing cycle, i.e. 21 days^1^. Also, the average within-flock prevalence (π) was calculated by averaging the proportion of infectious ducks across the infectious period of the flock. A total of 1000 Monte Carlo simulations of the model were run, initialised with 1999 susceptible ducks and one infectious duck, as the average size of a nomadic FGD flock in South Viet Nam ranges between 1,500^2^ and 2,200^1^. These Monte Carlo simulations were used to define the probability distributions for *g_cycle_* and π (Figure S1).


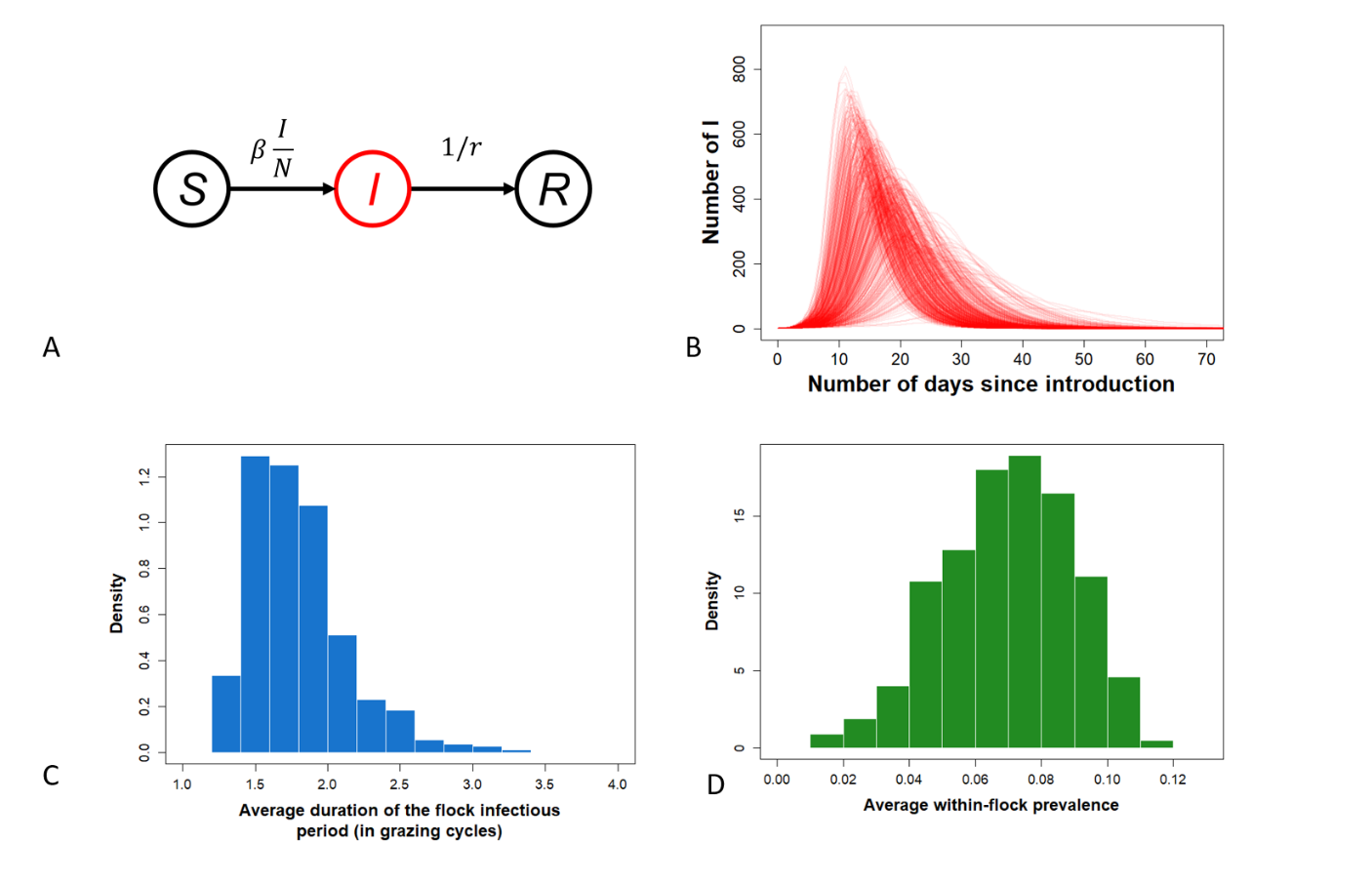


**Figure S1. Within-flock transmission model of highly pathogenic avian influenza virus H5N1 and simulation outputs.** A: schematic representation of the compartmental model structure (S: susceptible; I: infectious; R: recovered; N: total population; *β*: transmission rate assumed to be distributed according to a PERT(0.5, 0.8, 1.2); *r*: duration of the infectious period assumed to be distributed according to a PERT(2,3,4)); B: Simulated dynamics of the within-flock epidemic of HPAIV H5N1; C: distribution of the average duration of the flock infectious period (in number of grazing cycle); D: distribution of the average within-flock prevalence during the infectious period.

**References**

1. Meyer, A. *et al.* Movement and contact patterns of long-distance free-grazing ducks and avian influenza persistence in Vietnam. *PLOS ONE* **12**, e0178241 (2017).

2. Henning, J. *et al.* Characteristics of two duck farming systems in the Mekong Delta of Viet Nam: stationary flocks and moving flocks, and their potential relevance to the spread of highly pathogenic avian influenza. *Trop. Anim. Health Prod.* **45**, 837–848 (2013).
